# The ecological dichotomy of ammonia-oxidizing archaea and bacteria in the hyper-arid soils of the Antarctic Dry Valleys

**DOI:** 10.3389/fmicb.2014.00515

**Published:** 2014-09-30

**Authors:** Catarina M. Magalhães, Ana Machado, Béatrice Frank-Fahle, Charles K. Lee, S. Craig Cary

**Affiliations:** ^1^Interdisciplinary Centre of Marine and Environmental Research, University of PortoPorto, Portugal; ^2^Institute of Biomedical Sciences University of PortoPorto, Portugal; ^3^Institute of Groundwater Ecology, Helmholtz Zentrum München – German Research Center for Environmental HealthNeuherberg, Germany; ^4^International Centre for Terrestrial Antarctic Research, University of WaikatoHamilton, New Zealand; ^5^College of Earth, Ocean and Environment, University of DelawareLewes, DE, USA

**Keywords:** archaea, AOA, bacteria, AOB, ammonia oxidizers, Antarctica, Dry Valleys

## Abstract

The McMurdo Dry Valleys of Antarctica are considered to be one of the most physically and chemically extreme terrestrial environments on the Earth. However, little is known about the organisms involved in nitrogen transformations in these environments. In this study, we investigated the diversity and abundance of ammonia-oxidizing archaea (AOA) and bacteria (AOB) in four McMurdo Dry Valleys with highly variable soil geochemical properties and climatic conditions: Miers Valley, Upper Wright Valley, Beacon Valley and Battleship Promontory. The bacterial communities of these four Dry Valleys have been examined previously, and the results suggested that the extremely localized bacterial diversities are likely driven by the disparate physicochemical conditions associated with these locations. Here we showed that AOB and AOA *amoA* gene diversity was generally low; only four AOA and three AOB operational taxonomic units (OTUs) were identified from a total of 420 AOA and AOB *amoA* clones. Quantitative PCR analysis of *amoA* genes revealed clear differences in the relative abundances of AOA and AOB *amoA* genes among samples from the four dry valleys. Although AOB *amoA* gene dominated the ammonia-oxidizing community in soils from Miers Valley and Battleship Promontory, AOA *amoA* gene were more abundant in samples from Upper Wright and Beacon Valleys, where the environmental conditions are considerably harsher (e.g., extremely low soil C/N ratios and much higher soil electrical conductivity). Correlations between environmental variables and *amoA* genes copy numbers, as examined by redundancy analysis (RDA), revealed that higher AOA/AOB ratios were closely related to soils with high salts and Cu contents and low pH. Our findings hint at a dichotomized distribution of AOA and AOB within the Dry Valleys, potentially driven by environmental constraints.

## INTRODUCTION

Nitrification represents the oxidative part of the nitrogen (N) cycle and refers to the two-step process where ammonia is oxidized to nitrite and subsequently to nitrate. This process is considered a central biological pathway in the global N budget and productivity of terrestrial and aquatic ecosystems. From 1890 until 2004, scientists believed that only bacteria mediated aerobic ammonia oxidation. The recent discovery of a chemoautotrophic ammonia-oxidizing archaeon, *Nitrosopumilus maritimus* (Könneke et al., 2005), transformed our concept of the nature of organisms involved in nitrification, highlighting the importance of ammonia-oxidizing archaea (AOA) as potential participants in global biogeochemical N transformations (Hallam et al., 2006; Brochier-Armanet et al., 2008; de la Torre et al., 2008; Pester et al., 2012). The phylogenetic uniqueness of these archaea led to the creation of a novel archaeal phylum, *Thaumarchaeota*, comprising all AOA (Brochier-Armanet et al., 2008). The idea that nitrification activities from AOA greatly contribute to the global *N*-cycle is now generally accepted, and widespread distribution of AOA has been demonstrated (e.g., Francis et al., 2005; Biller et al., 2012; Stahl and de la Torre, 2012). Quantification of the relative abundances of AOA and ammonia-oxidizing bacteria (AOB) in different habitats (e.g., Leininger et al., 2006), including in Antarctic Peninsula soils (Jung et al., 2011), indicated a general dominance of AOA over AOB. However, ammonia-oxidizing betaproteobacteria have been shown to be more abundant and potentially more active than AOA in some estuarine and coastal sediments (Santoro et al., 2008; Magalhães et al., 2009), suggesting that the relative abundances and functional importance of AOB vs. AOA could vary in natural ecosystems. Recent studies indicated that environmental drivers like substrate (i.e., NH_4_^+^) concentration, pH, oxygen availability, salinity, among others, might be responsible for differentiating AOA and AOB abundance and distribution (Martens-Habbena and Stahl, 2011; Hatzenpichler, 2012; He et al., 2012; Prosser and Nicol, 2012; Zhalnina et al., 2012; Zhang et al., 2012). Despite previous attempts to evaluate drivers of natural AOA and AOB population dynamics, there remain large gaps in our understanding of factors that control AOA vs. AOB prominence in numerous ecosystems (Hatzenpichler, 2012; Prosser and Nicol, 2012; Monteiro et al., 2014).

The distribution and abundance of biota in the McMurdo Dry Valleys of Victoria Land in Antarctica are subject to strong spatial structuring due to the extreme heterogeneity in soil geochemical properties and severe climate gradients (Wood et al., 2008; Pointing et al., 2009; Lee et al., 2012; Magalhães et al., 2012). The dominance of environmental filters, together with the trophic simplicity of the ecosystem, makes Dry Valley soils a perfect model for investigating the physicochemical drivers of microbial biodiversity and function. In this study, we examined how environmental variables may determine the diversity and abundance of AOA and AOB *amoA* genes in the Dry Valleys. Although N is thought to be the limiting factor in many terrestrial Antarctic ecosystems, particularly in the Dry Valleys, little is known about the abundance and diversity of organisms and genes involved in the N cycle (Barrett et al., 2007; Hopkins et al., 2008; Cary et al., 2010; Niederberger et al., 2012). Studies of microbial N processes in the Dry Valleys have primarily focused on the *N*-fixation pathway. These studies demonstrated an unexpectedly high diversity of diazotrophs in Dry Valley soils (Cavacini, 2001; Wood et al., 2008; Pointing et al., 2009), suggesting that both cyanobacteria and a diverse range of heterotrophic diazotrophs are important players in the total input of N to these extreme oligotrophic environments (Niederberger et al., 2012). Interestingly, previous surveys of bacterial 16S rRNA genes in the Dry Valleys recovered sequences closely related to AOB groups (i.e., *Nitrosomonas*; Niederberger et al., 2008; Lee et al., 2012), and a GeoChip analysis of Dry Valley soils identified genes involved in the N cycle (Chan et al., 2013). However, there is very limited research on the dynamics of AOB and archaea in these hyper-arid cold deserts. Reports of bacterial and archaeal *amoA* abundance and diversity are so far restricted to the considerably wetter Antarctic Peninsula (Yergeau et al., 2007; Jung et al., 2011). Recent studies reporting limited diversity and abundance of Archaea in the Dry Valleys have identified a consistently high proportion of sequences (80–99%) affiliated with *Thaumarchaeota* (formerly known as *Crenarchaeota* Marine Group 1.1b; Ayton et al., 2010; Richter et al., 2014). These findings represent cursory evidence for archaeal nitrification in the Dry Valleys.

In this study, we investigated the distribution, abundance, and diversity of AOA and AOB *amoA* genes in four McMurdo Dry Valleys, where soil bacterial diversity and geochemistry have been previously described (Lee et al., 2012). The previous study reported a high degree of physicochemical heterogeneity and distinct bacterial communities, likely driven by the disparate physicochemical conditions. We hypothesized that such physicochemical heterogeneities exert similar selective effects on AOA and AOB *amoA* genes distribution and abundance.

## MATERIALS AND METHODS

### DRY VALLEYS SOIL SAMPLES COLLECTION

**FIGURE 1:**
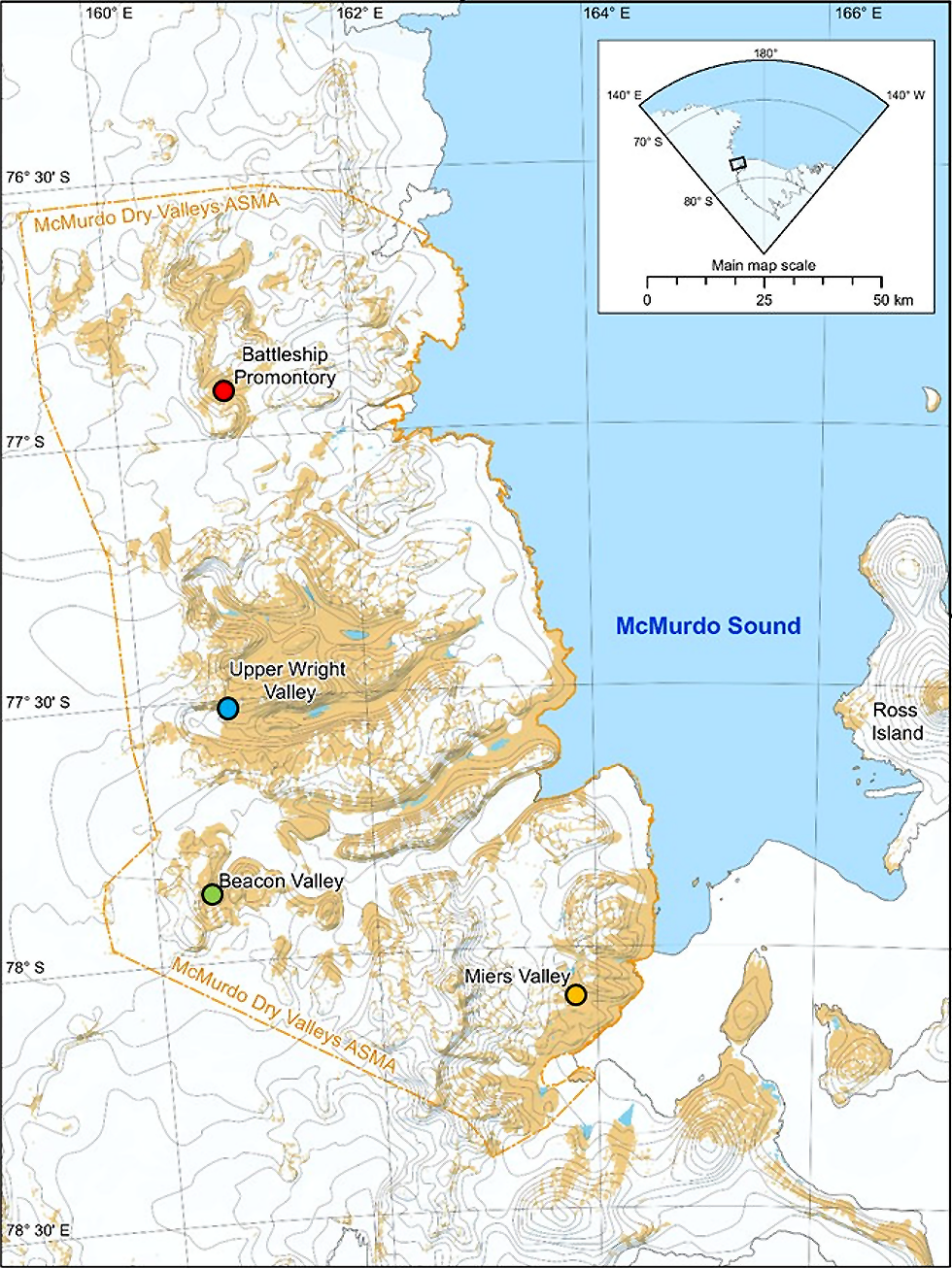
**Map of the McMurdo Dry Valleys and the sampling sites**.

Soils were collected from four different McMurdo Dry Valleys (**Figure 1**): Miers Valley (MV; 78°60’S 164°00’E), Upper Wright Valley (UW; 77°10’S, 161°50’E), Beacon Valley (BV; 77°48’S, 160°48’E), and Battleship Promontory (BP; 76°54’S 160°55’E). Miers Valley is a coastal, low altitude valley (153 m) with comparatively high C/N ratio and has been noted for sustaining diverse cyanobacterial and bacterial communities (Wood et al., 2008; Lee et al., 2012). Beacon and Upper Wright Valleys are higher altitude valleys (1500 and 1000 m, respectively), characterized by extremely low temperatures, strong desiccating winds, low C/N ratios, and high soil electrical conductivity, creating comparatively inhospitable environments for soil microorganisms (Wood et al., 2008; Lee et al., 2012). Battleship Promontory is a high altitude valley (1000 m) with transiently liquid water in snow melt ponds, leading to lower soil electrical conductivity and higher moisture content and creating favorable conditions for bacterial communities (Lee et al., 2012).

In December 2006 (Miers Valley and Beacon Valley) and January 2008 (Battleship Promontory and Upper Wright Valley), two perpendicular transects of 50 m intersecting in the center were laid out at each sampling site, and four sampling points (A–D) were taken at the ends of each transect (Lee et al., 2012). At each sampling site, an area of 1 m^2^ was identified, and one scoop of soil was collected aseptically from the top 2 cm at the four corners of this 1 m^2^ area and combined in a sterile Whirl-Pak (Nasco International Inc., Fort Atkinson, WI, USA). All necessary and appropriate precautions were taken to avoid anthropogenic or cross-site contaminations. Samples were stored at -20°C at the earliest opportunity and transported back to University of Waikato, New Zealand, where they were stored at -80°C until analysis. Soil geochemical properties were determined as previously described (Lee et al., 2012).

### DNA EXTRACTION

From each of the four samples collected at a sampling site (A–D), six replicates of DNA were extracted (total of 24 extractions per valley) from 0.6 to 0.8 g of homogenized soil using a modification of the CTAB (bromide-polyvinylpyrrolidone-bmercaptoethanol) extraction protocol (Coyne et al., 2001; Barrett et al., 2006). Recovered DNA was stored at –80°C. Reproducibility of DNA recovery, quantified using the QuBit dsDNA HS Kit (Life Technologies, Portugal), was tested among the six replicates (coefficient of variation = 14%). Three of the DNA extraction replicates were combined to obtain sufficient amounts of DNA for molecular analyses. Internal variations for each sample were assessed by processing two pooled replicates (composed of three DNA extractions) for each soil sample.

### CLONING AND PHYLOGENETIC ANALYSIS

To assess AOA and AOB *amoA* genes diversity, two soil samples from each Dry Valley (i.e., MV-A, MV-B, BP-A, BP-B, BV-A, BV-B, UW-A, and UW-B) were randomly selected for clone library construction. Archaeal and bacterial *amoA* genes were amplified using Arch-amoAR/Arch-amoAF (Francis et al., 2005) and amoA-1F/534R (Rotthauwe et al., 1997) primers, respectively (**Table 1**). PCR products, generated using a previously described PCR protocol (Magalhães et al., 2009), were visualized with agarose gel (1.5%) electrophoresis, and a band of appropriate size was excised and purified with the QIAquick Gel Extraction Kit (Qiagen, Portugal). The resulting PCR amplicons were cloned using the TOPO TA Cloning Kit (Life Technologies, Portugal) according to manufacturer instructions. Plasmids were isolated using the GeneElute Plasmid Miniprep Kit (Sigma Aldrich, Spain), and DNA concentrations were determined with the QuBit dsDNA BS kit (Life Technologies, Portugal). Thirty colonies were selected randomly from each clone library (i.e., a total of 480 colonies for AOA and AOB *amoA* genes), and their insert sizes were verified by digesting 0.6–0.8 μg of DNA at 37°C for 2h with 10 U of *EcoRI* (Sigma-Aldrich, Portugal). A total of 420 clones with the correct insert size and containing likely AOA and AOB *amoA* genes were screened using restriction fragment length polymorphism analysis, where 0.6–0.8 μg of DNA was digested with 10 U of *MspI* (Promega, Europe) at 37°C overnight. A total of 15 clones of *amoA* AOA and AOB genes from each valley (total 120) were selected for sequencing in the STABVIDA Sequencing Facilities (Lisbon, Portugal).

**Table 1:** Primers used in this study.

Target gene	Primers	Sequence (5′–3′)	Reference
16s rRNA	341F	CCT ACG GGA GGC AGC AG	Muyzer et al. (1993)
	534R	ATT ACC GCG GCT GCT GG	
βAOB *amoA*	amoA-1F	GGG GTT TCT ACT GGT GGT	Rotthauwe et al. (1997)
	amoA-2R′	CCT CKG SAA AGC CTT CTT C	Okano et al. (2004)
Archaea *amoA*	Arch -amoAF	STAATGGTCTGGCTTAGACG	Francis et al. (2005)
	Arch - amoAR	GCGGCCATCCATCTGTATGT	Francis et al. (2005)
qPCR-AOA	AOAF	CCTACCACAAGCATAGT	This study
	AOAR	GTTAACAGCACCTTACTTACT	This study
qPCR-AOB	AOBF	GTCTCCATGCTCATGTTC	This study
	AOBR	GGACCTTTGACGTAGAAGAA	This study

Sequences were aligned with published AOA and AOB *amoA* sequences in GenBank using the basic local alignment search tool (BLAST). All sequences were aligned with Clustal W (Thompson et al., 1994) as implemented in Bioedit version 7.0.5 (Hall, 1999), and phylogenetic trees were constructed using MEGA version 3.1 (Kumar et al., 2001) with both maximum parsimony and neighbor-joining methods. Bootstrap analysis (1,000 replicates) was carried out. We used a 98% similarity cut off for defining operational taxonomic units (OTUs). Clone sequences from this study have been deposited in GenBank under accession numbers KF574112 to KF574224.

### QUANTITATIVE REAL-TIME PCR

Quantitative PCR (qPCR) was conducted in a CFX96 real-time PCR detection system (Bio-Rad, Portugal) to determine copy numbers of bacterial 16S rRNA, bacteria *amoA*, and archaeal *amoA* genes using previously described 16S rRNA primers and new *amoA* primers designed for this study (**Table 1**). For each soil sample, qPCR was performed in triplicates for both pooled DNA replicates as 25 μl reactions with 10–20 ng of template DNA in each reaction. Each qPCR tube (white 0.2 ml PCR strips with ultra clear optical flat caps, [Bio-Rad, Portugal]) contained 12.5 μl of iQ Sybr Green Supermix (BioRad, Portugal), 2 μl of each primer (10 μM), and nuclease-free water (Promega, Portugal). For both primer sets, the thermal cycler was programmed for 5 min of denaturation at 94°C; followed by 8 cycles of denaturation at 94°C for 30 s, annealing at 65°C for 30 s, and extension at 72°C for 30 s; followed by 27 cycles where the annealing temperature was changed to 57 °C; and a final extension step at 72°C for 10 min. Standards consisted of plasmids with AOA and AOB *amoA* gene inserts from clones generated for this study, as described above. Standard curves were generated in duplicate for each primer set, and amplification of standards was linear over six orders of magnitude (i.e., 0.2–0.2 × 10^-6^ ng of DNA). The *R^2^* values between plasmid DNA copy numbers and the calculated threshold cycle values ranged from 0.98 to 1.00, and amplification efficiency ranged between 98 and 101% for all standard curves. Target copy numbers were calculated with an average molecular weight of 618 g mol^-1^, and data were standardized to copies of gene per g of sediment. Melting curves and agarose gel electrophoresis of the qPCR products were carried out following each qPCR assayed to confirm identity of the PCR products. Primers for qPCR were designed for AOA and AOB (**Table 1**) using AlleleID 7.6 software (Prenier Biosoft, International).

### STATISTICAL ANALYSES

Relationships between gene copy numbers and soil physicochemical properties (described in Lee et al., 2012) for 16 samples (four sampling sites in each of the four Dry Valleys) were analyzed using multivariate ordination tools. Redundancy analysis (RDA) was selected as the preferred ordination method (ter Braak and Smilauer, 2002) and performed using CANOCO (version 4.5, Microcomputer Power, Ithaca, NY, USA). For RDA, environmental variables (i.e., pH, electrical conductivity, gravimetric water content, C/N, Mg, Cr, Mn, Co, Ni, and Cu) were normalized to a mean of 0 and SD of (1). Monte Carlo permutation test was used to assess the statistical significance of the relationships.

## RESULTS AND DISCUSSION

### AOA AND AOB DIVERSITY

**FIGURE 2:**
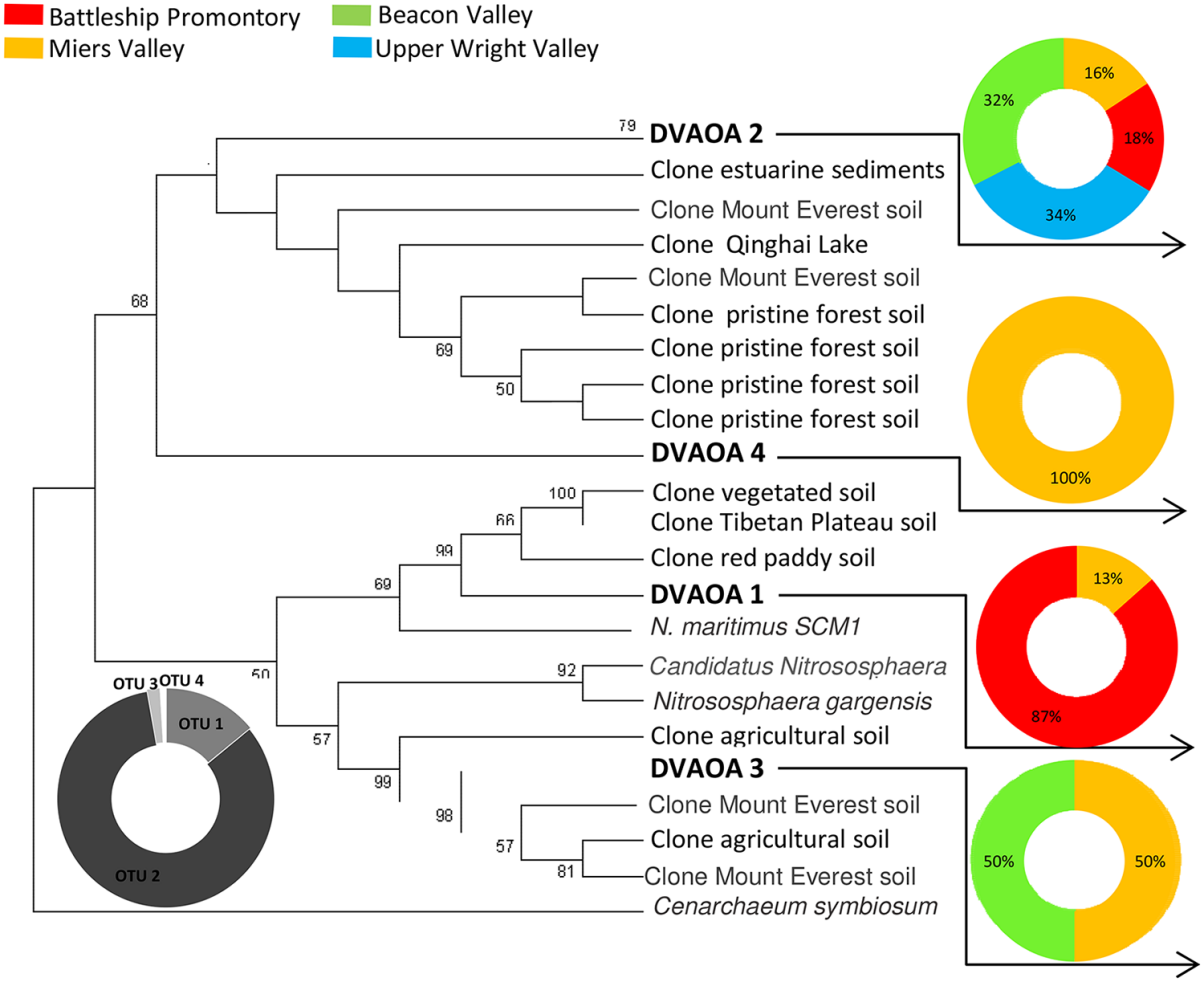
**Phylogenetic relationship among Archaeal *amoA* sequences retrieved from Antarctica Dry Valleys and other geographic locations.** The neighbor-joining tree was based on 591 nucleotide sequences and was constructed based on Kimura distances and the neighbor-joining method. Distance bootstrap values ≥50% are indicated at branch points (1000 iterations). The major clusters indicated were also supported by maximum parsimony analysis. The 210 Archaeal *amoA* clones obtained from this study are distributed under DVAOA1, DVAOA2, DVAOA3, and DVAOA4. Gray scale pie plot indicate the percentage of occurrence of each OTU in the total sequences, and colorful pie plots indicate the percentage of occurrence of each OTU within the four Dry Valleys.

**FIGURE 3:**
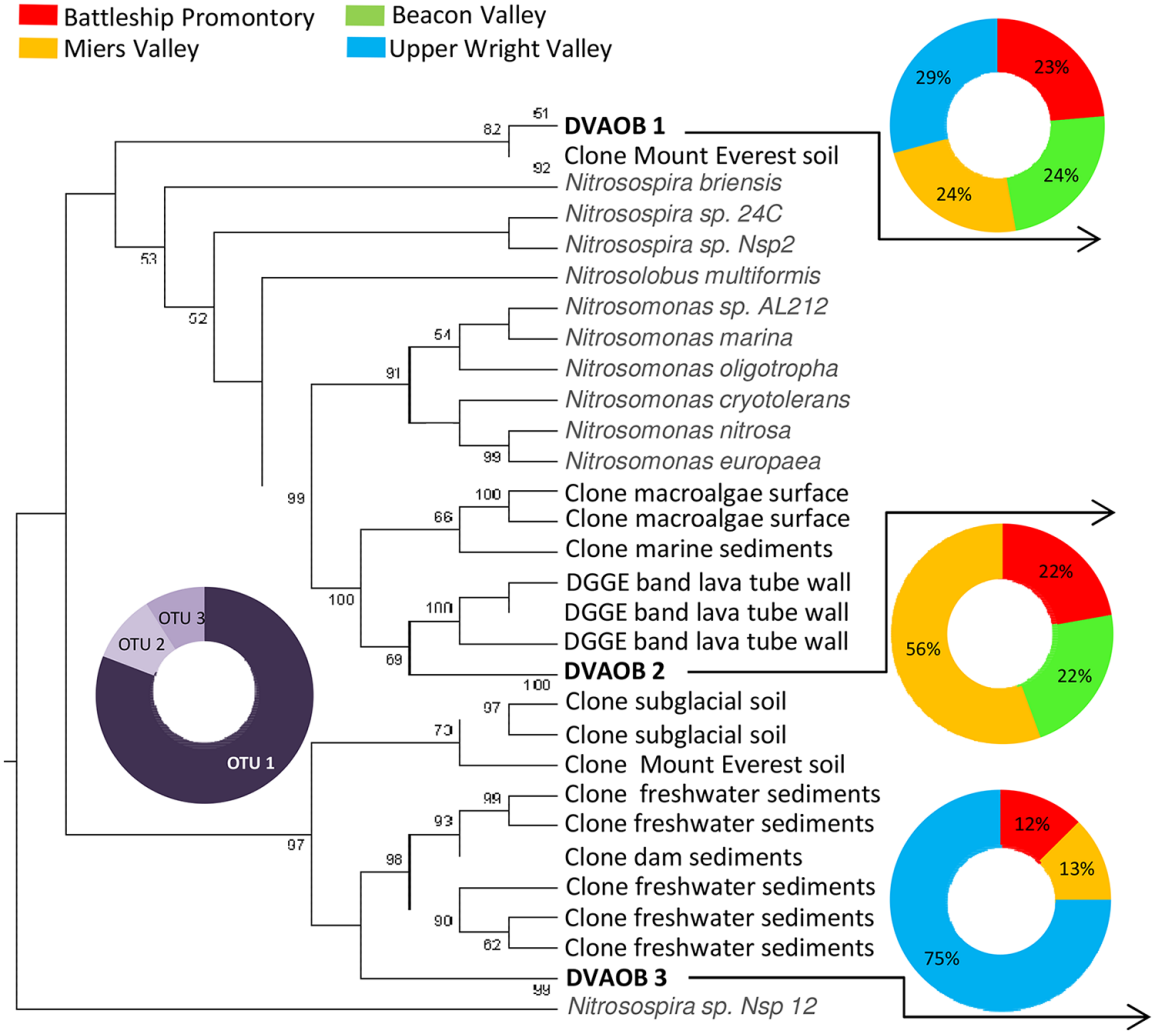
**Phylogenetic relationship among Bacterial *amoA* sequences retrieved from Antarctica Dry Valleys and other geographic locations.** The neighbor-joining tree was based on 443 nucleotide sequences and was constructed based on Kimura distances and the neighbor-joining method. Distance bootstrap values ≥50% are indicated at branch points (1000 iterations). The major clusters indicated were also supported using maximum parsimony analysis. The 210 Bacterial *amoA* clones obtained from this study are distributed under DVAOB1, DVAOB2, and DVAOB3. Gray scale pie plot indicate the percentage of occurrence of each OTU in the total sequences, and colorful pie plots indicate the percentage of occurrence of each OTU within the four Dry Valleys.

Previous studies have reported high diversities and widespread distribution of AOA and AOB in natural and managed soils, marine and estuarine water and sediments, wastewater treatment bioreactors, hot springs, and many other environments (Francis et al., 2005; Beman and Francis, 2006; Leininger et al., 2006; Park et al., 2006; Dang et al., 2008; Magalhães et al., 2009; Biller et al., 2012; Stahl and de la Torre, 2012). In this study, we report AOA and AOB presence in the extreme environments of the Transantarctic Mountains. AOA and AOB *amoA* gene diversity recovered from these soils were extremely low, with only four AOA and three AOB *amoA* OTUs identified from a total of 420 clones (**Figures 2 and 3**). However, we cannot exclude the possibility that our primers failed to amplify some *amoA* genes, resulting in a lower observed AOA and AOB *amoA* diversity since considerable numbers of microorganisms in the Dry Valleys cannot be reliable assigned to higher taxonomic levels (Cary et al., 2010).

Archaea were only recently successfully detected in the Ross Sea region (Ayton et al., 2010) and across the McMurdo Dry Valleys (Richter et al., 2014), with reportedly low diversity and dominance of certain OTUs in these harsh environments. In Antarctic coastal mineral soils, 99% of the detected 16S rRNA sequences were affiliated with *Thaumarchaeota* (Ayton et al., 2010), as did more than 80% of all archaeal sequences identified in the McMurdo Dry Valleys (Richter et al., 2014). These findings hint at a high prevalence of *amoA* within the archaeal communities of Antarctic Dry Valley soils, which have been demonstrated by our results to have low genetic diversity (**Figure 2**). Taking this into account the quantification of the *amoA* could be a good representative of the whole archaeal community inhabiting Dry Valley soils.

Across the four AOA OTUs identified (DVAOA 1–4), 89% of the archaeal *amoA* clone sequences fell within DVAOA 2, which was also the only OTU recovered from all four sampling sites (**Figure 2**). The representative sequence of DVAOA 2 showed 95–96% nucleotide sequence similarity to sequences retrieved from a wide range of environments, such as temperate oxic lake water and anoxic sediments (Jiang et al., 2009), sediments from Plum Island Sound estuary [(Boston, Massachusetts, USA) Bernhard et al., 2010], alpine and permafrost Mount Everest bare soils (Zhang et al., 2009) and temperate pristine forest soils (Szukics et al., 2010). DVAOA2 is also less than 80% similar to the *amoA* gene of any known AOA isolates [71% to *N. maritimus* (Könneke et al., 2005)] and 79% to *Candidatus Nitrososphaera gargensis* (Spang et al., 2012). Only one AOA *amoA* OTU (DVAOA 2) was identified in the Upper Wright Valley, and Miers Valley had the highest diversity of *amoA* AOA, with four different OTUs present (**Figure 2**).

The identified AOB *amoA* sequences fell into two distinct phylogenetic clusters (**Figure 3**). All three *amoA* AOB OTUs (DVAOB1-3) were present in Miers Valley, whereas the other Dry Valleys contained only two of those OTUs. Similarly to the pattern for AOA *amoA*, the majority (81%) of *amoA* AOB clones fell within one OTU (i.e., DVAOB1), and the remaining clones (19%) were equally distributed between DVAOB2 and DVAOB3 (**Figure 3**). The representative sequences for DVAOB1 are 97% similar to a AOB *amoA* sequence detected in alpine soils on Mount Everest (Zhang et al., 2009). It is worth noting that a higher AOB *amoA* gene diversity (with sequences within four *Nitrosospira* and two *Nitrosomonas*-like clusters) was reported for the Mt. Everest soils (Zhang et al., 2009). Representative sequences for DVAOB1 and DVAOB2 were more closely related to *Nitrosospira*-like entries in GenBank (between 87 and 89% similarity) than *Nitrosomonas*-like sequences (between 70 and 78% similarity). Interestingly, previous studies have demonstrated that *Nitrosomonas*-like species are more tolerant of high environmental NH_4_^+^ concentrations than *Nitrosospira*-like species (de Bie et al., 2001; Cébron et al., 2004; Caffrey et al., 2007; Magalhães et al., 2009), which are typically associated with pristine environments with low NH_4_^+^ concentrations (Stephen et al., 1996, 1998; McCaig et al., 1999). In the extremely oligotrophic Dry Valley soils, nutrient limitation has been found to impose strong limitations on the distribution of soil microbiota (Cary et al., 2010; Lee et al., 2012; Magalhães et al., 2012). The representative sequence of DVAOB 3 showed lower similarities to *amoA* gene sequences from isolates (73–77% similarity). However, clustered (94–95% similarity) with sequences retrieved from a range of contrasting environments like lava cave walls biofilms (Azores, Portugal (Hathaway et al., 2014), sediments from subtropical freshwater marshes [Hongne Reserve, China (Lee and Gu, unpublished)] and subglacial soils from Robertson Glacier [Alberta, Canada (Boyd et al., 2011)].

Our findings showed that although AOA and AOB *amoA* gene diversities in the Dry Valleys were low, there were clear differences in the distribution of AOA and AOB among the four Dry Valleys examined.

### AOA AND AOB COMMUNITY ABUNDANCE

**FIGURE 4:**
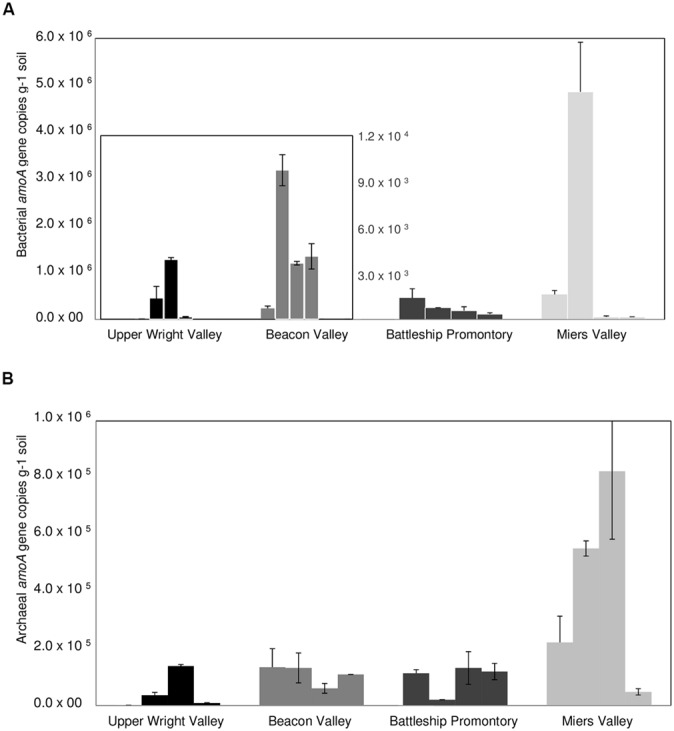
**Bacterial **(A)** and archaeal **(B)***amoA* gene copy numbers for locations A, B, C, and D) in Upper Wright, Beacon and Miers Valleys and in Battleship Promontory (mean ± SD of 3 qPCR replicates)**.

**FIGURE 5:**
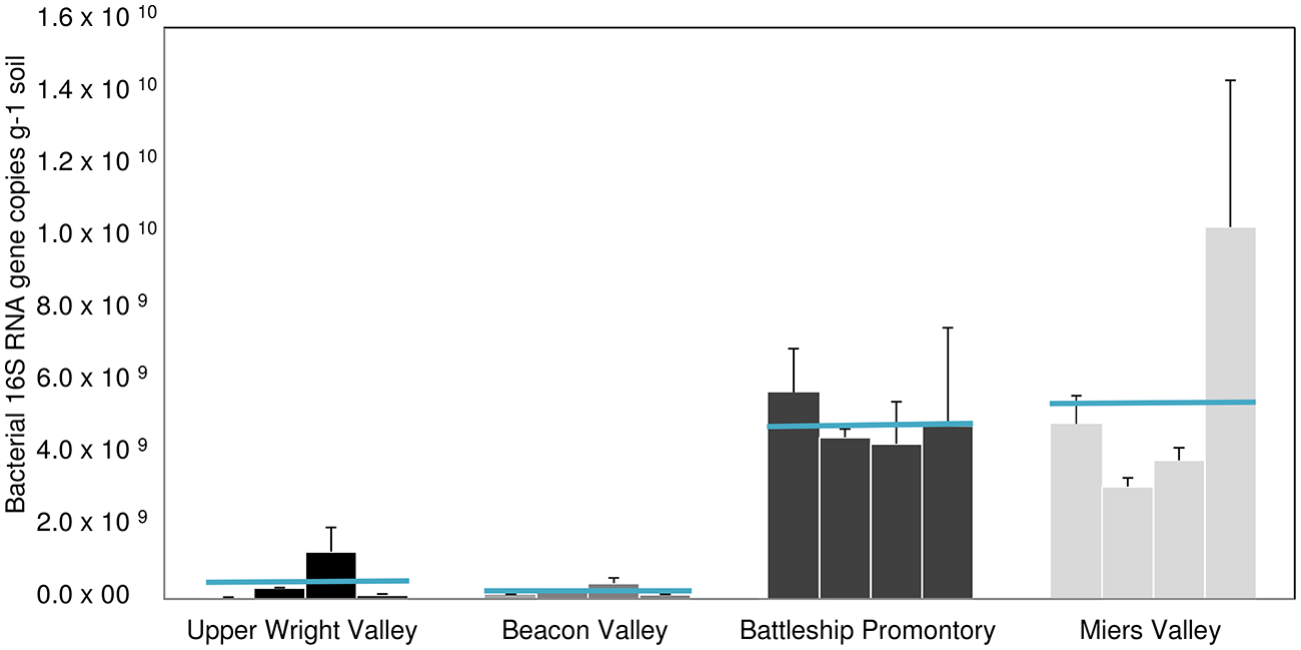
**16S rRNA gene copy numbers for locations A, B, C, and D of the four Dry Valleys (mean ± SD of 3 qPCR replicates).** Blue lines show the mean value for the four sites of each valley.

qPCR analysis of AOB *amoA* gene showed substantially higher abundance of AOB *amoA* gene at BP and MV (**Figure 4A**) compared to UW and BV (ANOVA, *p* < 0.001; **Figure 4A**). Coincidentally, qPCR analysis of bacterial 16S rRNA genes showed much higher abundance in BP and MV (5.6 ± 1.4 × 10^9^ and 4.9 ± 1.4 × 10^9^ copies g^-1^ soil, respectively) than in UW and BV (2.2 ± 1.4 × 10^8^ and 4.4 ± 1.8 × 10^8^, copies g^-1^ soil, respectively; **Figure 5**). These values fall within the range of bacterial 16S rRNA gene copy numbers previously reported for McKelvey Valley (0.06 × 10^8^ to 2.4 × 10^8^, Pointing et al., 2009), and for Ross Island and several McMurdo Dry Valleys, from direct cell counts (Ayton et al., 2010). AOA *amoA* gene qPCR results showed a different pattern, where *amoA* gene copy numbers did not differ significantly among samples from UW, BV, and BP, and were only significantly higher (*p* < 0.001) in samples from MV (**Figure 4B**). Soils from MV have been described as geochemically distinct (i.e., high C/N, high pH, etc.; Lee et al., 2012) from other Dry Valleys, and these conditions may favor nitrification and thus allow high diversity and abundance of bacterial and archaeal *amoA*. This is congruent with high bacterial and cyanobacterial diversities previously documented in samples from MV (Wood et al., 2008; Lee et al., 2012).

**FIGURE 6:**
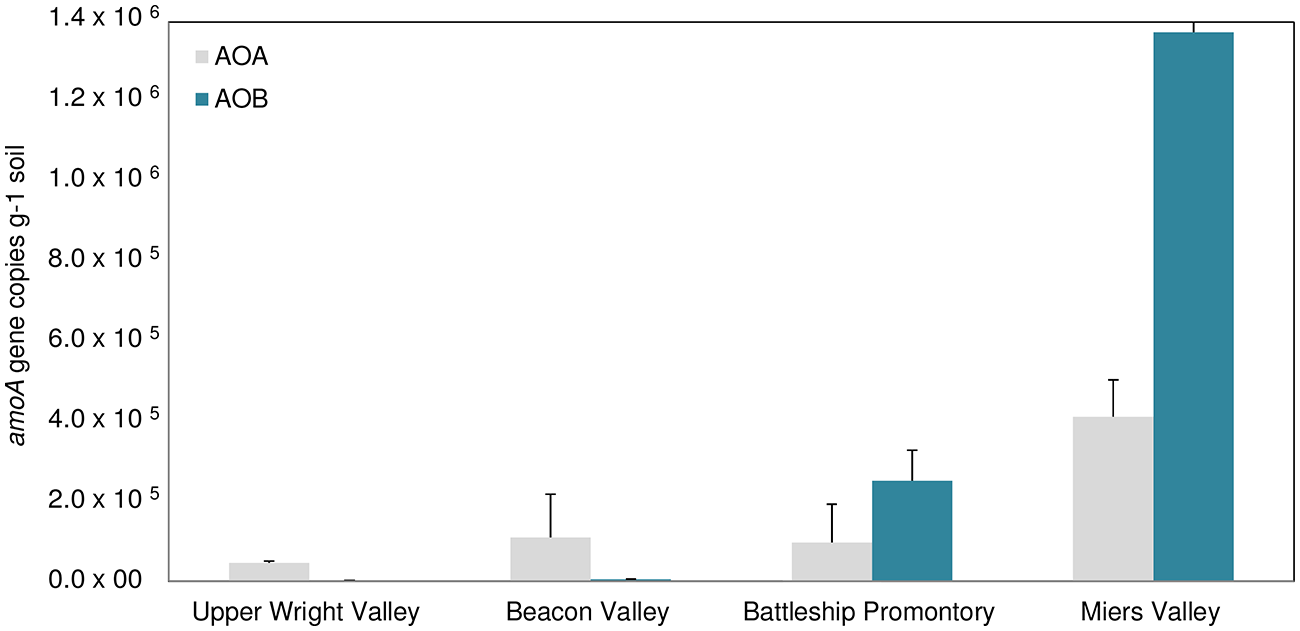
**Bacterial vs. archaeal *amoA* genes copy numbers in Upper Wright, Beacon and Miers Valleys and in Battleship Promontory (mean ± SD, of 3 qPCR replicates performed in each of the four sites sampled in each valley, *n* = 12)**.

Large spatial differences in AOA and AOB *amoA* gene abundance were detected among the four Dry Valleys (**Figures 4 and 6**). For example, AOB *amoA* gene abundances were three orders of magnitude higher in MV soils relative to UW soils (**Figures 4A and 6**). AOA and AOB *amoA* gene abundance reported here are similar to values obtained in higher altitude soils (≥5700 m) of Mount Everest, but one to three magnitudes lower than those observed for lower elevation mountain soils (<5400 m; Zhang et al., 2009). Interestingly, the *amoA* gene copy numbers for the Dry Valleys are significantly higher than those reported for soils from the Antarctic Peninsula (Jung et al., 2011). This may be due to the fact that we developed and optimized a new set of qPCR primers for archaeal and bacterial *amoA* genes found in the Dry Valleys, whereas the qPCR primers used by Jung et al. (2011) were based on existing primer sets developed for very different environments (Rotthauwe et al., 1997; Okano et al., 2004; Francis et al., 2005; de la Torre et al., 2008).

Since the discovery of chemoautotrophic AOA (Venter et al., 2004; Könneke et al., 2005; Schleper et al., 2005; Treusch et al., 2005), their high relative abundances have been reported for many systems (Hallam et al., 2006; Leininger et al., 2006; Wuchter et al., 2006; Nicol et al., 2008). These studies showed a general prevalence of AOA over AOB, as was previously reported for soils from the Antarctic Peninsula (Jung et al., 2011). We observed more complex AOA vs. AOB dynamics in the Dry Valleys, with significant differences in the four Dry Valleys examined. Although AOB outnumbered AOA in MV and BP, high relative abundances of AOA *amoA* genes were found in UW and BV (**Figure 6**), where lower bacterial 16S rRNA gene abundance (**Figure 5**) and diversity have been described (Lee et al., 2012). Quantitative PCR analysis of *amoA* genes revealed clear differences in the relative abundances of AOA and AOB *amoA* genes among samples from the four Dry Valleys. A shift in the relative abundance of AOA and AOB has been previously reported for Mount Everest alpine and permafrost environments with greater abundance of AOA over AOB at lower (4000 m), and the reverse at higher (5800–6000 m) altitudes (Zhang et al., 2009). In addition, the results for MV and BP are in agreement with what had been observed in subglacial soils (Boyd et al., 2011), where bacteria appeared to be the predominant nitrifiers over archaea. It is also interesting to note that the AOB/AOA *amoA* gene ratio varied between 2.6 and 3.3 in MV and BP, but in UW and BV, where AOA *amoA* was more dominant, AOA/AOB ratios were much higher (33.1–24.0). Collectively, these results indicate spatial heterogeneity in the microorganisms that mediate the ammonia oxidation pathway in these extreme environments, and that the harsh conditions in UW and BV potentially impose physiological challenges for AOB and limit their distribution.

### ENVIRONMENTAL CONTROLS ON AOA AND AOB DISTRIBUTION

The clearly dichotomized distribution of AOA and AOB in the four Dry Valleys examined (**Figure 6**) provides a rare opportunity to identify the environmental factors that control the relative abundance and diversity of these two groups of ammonia oxidizers.

As previously described, soil geochemical characteristics of McMurdo Dry Valleys varied significantly among the studied valleys (Lee et al., 2012). Large heterogeneities in bacterial diversity (Lee et al., 2012), bacterial abundance (Pointing et al., 2009), and the diversity of stress response pathways (Chan et al., 2013) among different Dry Valleys reflect significant variances in soil geochemical properties that characterize the Dry Valley ecosystem (Bockheim, 1997; Lamsal and Paudyal, 1999; Lee et al., 2012). Among the Dry Valleys examined for this study, MV stood out by virtue of the high C/N ratio, high total % C content, low conductivity, and higher pH of its soils (Lee et al., 2012), which may explain higher overall AOA and AOB *amoA* gene copy numbers (**Figures 4A,B**) and diversity (**Figures 2 and 3**). The low C/N ratios and high soil conductivity that characterized BV and UW soils (Lee et al., 2012) correlate with lower bacterial abundance (**Figure 5**) and drastically lower AOB *amoA* diversity (**Figure 3**) and gene copy numbers (ANOVA, *p* < 0.01; **Figure 4A**).

**FIGURE 7:**
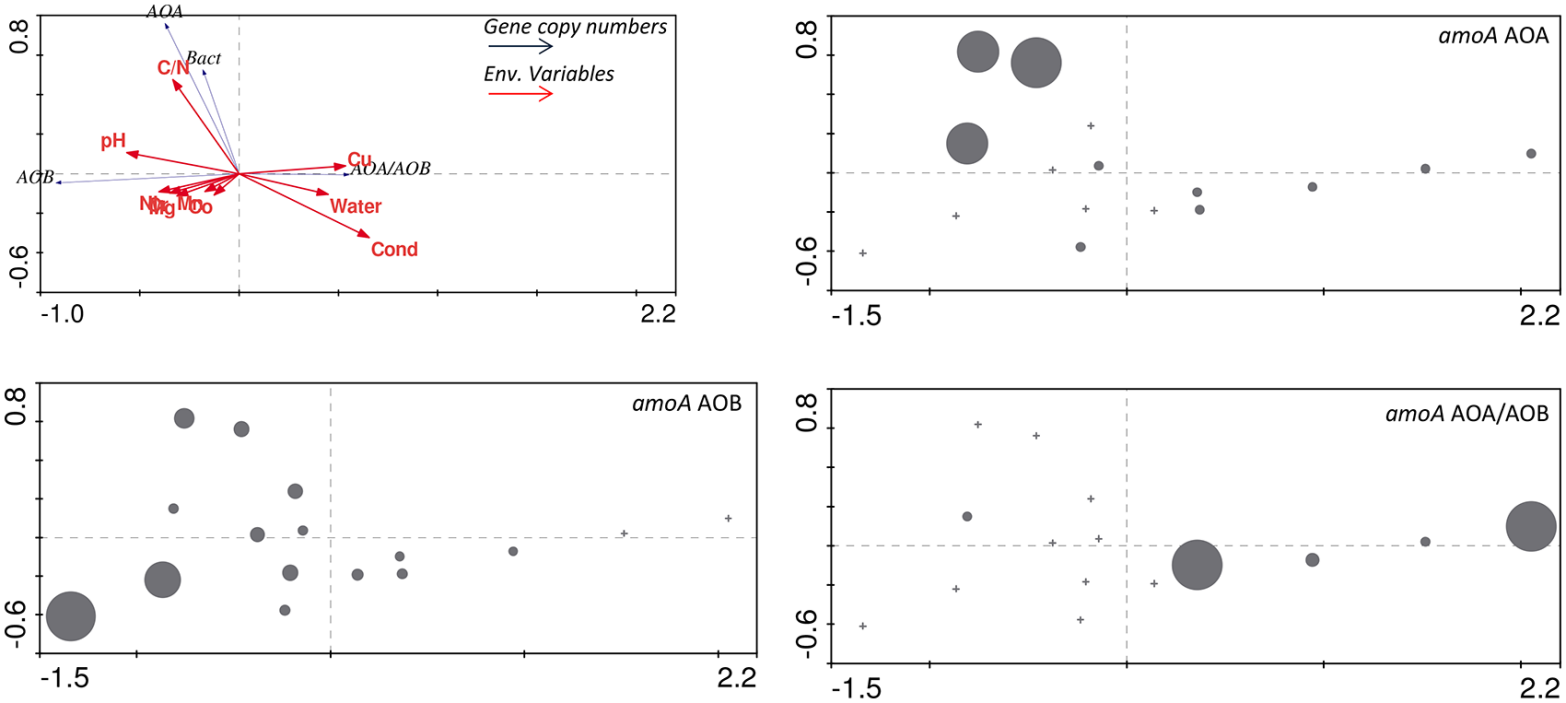
**Redundancy analysis ordination (RDA) plot for the biogeochemical variables and copy numbers of AOA and AOB *amoA* genes.** Gene copy values for AOA, AOB and ratios of *amoA* AOA/AOB are represented as circles of diameter scaled linearly to the magnitude of the value. In the RDA ordination diagram, the angle and length of the arrow relative to a given axis reveals the extent of correlation between the variable and the canonical axis (environmental gradient).

Correlations between environmental variables and 16S rRNA and *amoA* gene copy numbers were examined using (RDA; **Figure 7**). A Monte Carlo test of F-ratios (*F* = 2.54 and *p* = 0.044) identified ten environmental variables (pH, conductivity, gravimetric water content, C/N, Mg, Cr, Mn, Co, Ni, and Cu) among those reported in Lee et al. (2012) that significantly contributed to differences in gene copy numbers. The first gradient (RDA 1, horizontal, **Figure 7**) explained 75% of the variability in gene copy numbers and was highly correlated with the environmental variables (93.2%). The results revealed that higher abundance of AOA and AOB *amoA* genes are most strongly correlated with higher C/N ratio and pH in the soils (**Figure 7**). Higher AOA/AOB ratios were related to higher conductivity, Cu contents, and higher gravimetric water content (**Figure 7**). Our results suggest that AOA communities can better tolerate higher conductivity in BV and UW soils. This is in agreement with the documented prevalence of AOA over AOB in ocean waters (Santoro et al., 2010) where salinities are fairly stable at about 35 ppt. However, studies of coastal and estuarine sediments, characterized by highly fluctuating salinity levels, showed that high salinities favored numerical dominance of AOB over AOA (Santoro et al., 2008; Magalhães et al., 2009). Substrate (i.e., NH_4_^+^) concentration might also play an important role in the abundance and distribution of AOA and AOB, with AOA generally dominant in low NH_4_^+^ and oligotrophic environments (Martens-Habbena et al., 2009; Santoro et al., 2010; Martens-Habbena and Stahl, 2011; Zhalnina et al., 2012).

## CONCLUSION

The findings of this study represent the first report of the diversity and abundance of AOA and AOB communities in the Transantarctic Mountains, and make substantial contributions to our knowledge of the poorly understood N cycle of Antarctic Dry Valley soils. We confirmed the presence of archaeal and bacterial *amoA* genes in four Dry Valleys with disparate soil geochemical characteristics, demonstrating wide distributions and spatial heterogeneities of AOA and AOB, highlighting the potential role of nitrification in microbial processes in the Dry Valleys. We also identified a dichotomized distribution of AOA and AOB in the Dry Valleys that is potentially driven by environmental heterogeneities. Although more detailed studies are needed to fully understand the environmental drivers that control the relative abundance of AOA and AOB in natural environments, our results indicate that soil conductivity, may play an important role.

## Conflict of Interest Statement

The authors declare that the research was conducted in the absence of any commercial or financial relationships that could be construed as a potential conflict of interest.
